# Quality of Life and Related Paraclinical Factors in Iranian Patients with Transfusion-Dependent Thalassemia

**DOI:** 10.1155/2021/2849163

**Published:** 2021-08-18

**Authors:** Mehran Khodashenas, Parham Mardi, Nooshin Taherzadeh-Ghahfarokhi, Bahareh Tavakoli-Far, Mahnaz Jamee, Niloofar Ghodrati

**Affiliations:** ^1^Student Research Committee, Alborz University of Medical Sciences, Karaj, Iran; ^2^Dietary Supplements and Probiotic Research Center, Alborz University of Medical Sciences, Karaj, Iran; ^3^Department of Physiology and Pharmacology, School of Medicine, Alborz University of Medical Sciences, Karaj, Iran; ^4^Hematologist-Oncologist, Faculty Member, Department of Internal Medicine, Alborz University of Medical Sciences, Karaj, Iran

## Abstract

**Background:**

Thalassemia is one of the most common genetic hematologic disorders in the world. Despite outstanding achievements in prenatal diagnosis and a decrease in the number of patients, thalassemia is still a significant issue in most parts of the world, especially in the Mediterranean countries. Understanding the factors associated with this condition is crucial to help clinicians and policymakers provides social and medical support for patients to facilitate their lives. This study aims to appraise the quality of life (QoL) and its related paraclinical factors in Iranian transfusion-dependent thalassemia patients.

**Methods and Materials:**

This study is a cross-sectional study performed in the thalassemia clinic of Imam-Ali Hospital, Karaj, Iran. The demographic, clinical, and laboratory data of 100 patients with transfusion-dependent thalassemia were recorded. The patients' QoL was measured by the World Health Organization Quality of Life Instruments Brief (WHOQOL-BREF) version questionnaire. The results were analyzed using SPSS software.

**Results:**

This study demonstrated that all four features of life are influenced in transfusion-dependent thalassemia patients. Also, higher educational status and lower serum ferritin levels were associated with better scores in assessing the QoL. On the other hand, an elevated level of AST (aspartate transaminase), ALT (alanine transaminase), and FBS (fasting blood sugar) are associated with lower scores.

**Conclusion:**

All features of QoL are correlated to the patients' laboratory findings. Our data suggest that managing patients' laboratory indices is attributed to their higher QoL. We also suggest regular screening of patients' QoL to manage disease complications more efficiently.

## 1. Introduction

Thalassemia is an inherited blood disorder and one of the most common monogenic diseases in the world. It has been a critical health issue in the Mediterranean region, Middle East, Southeast Asia, and India [1, 2]. Overall, approximately 200 million people are affected globally [3]. Recent studies suggest that despite improvements in prenatal diagnosis, between 300,000 and 400,000 neonates are born with thalassemia every year, most of them in developing countries [4–7]. In Iran, the prevalence of thalassemia ranges from 1% to 10% in various parts of the country [1, 8]. It should be noted that about two million thalassemia carriers are registered in the country, making the disease a significant public health issue [9, 10]. Thalassemia is more prevalent in the northern and southern parts of the country [10, 11].

There are four types of thalassemia: *α*, *β*, *γ*, and *δ* thalassemia. *ß*-Thalassemia is a chronic autosomal recessive disorder in which *ß*-globin chain synthesis is decreased or absent. There are about 200 mutations associated with the thalassemia phenotype [12, 13]. In *ß*-thalassemia minor, the synthesis of hemoglobin A (*α*2, *β*2) decreases due to a single mutation in *ß*-gene, leading to red blood cell (RBC) destruction. While some *ß*-thalassemia patients are usually asymptomatic and only present with mild to moderate microcytic anemia [14], some patients require repeated blood transfusions. If patients' hemoglobin levels range from 7 to 10 mg/dl, they will only need occasional blood transfusions; while in cases with lower hemoglobin levels, regular blood transfusion is indicated [15].

These patients have a broad spectrum of clinical manifestations. For instance, ineffective erythropoiesis may cause skull and face deformity and lead to pathologic fractures. Moreover, chronic anemia increases iron absorption, which leads to iron overload. Iron overload causes severe complications in different organs' functions, including cardiac failure and endocrine abnormalities such as diabetes mellitus, hypogonadism, hypothyroidism, hypoparathyroidism, and growth hormone deficiency [16].

There is no suitable definite treatment for thalassemia. Management of patients includes blood transfusion to compensate for insufficient erythropoiesis and anemia. Iron chelators such as deferoxamine were administered, and splenectomy is indicated to reduce iron overload. It should be noted that while bone marrow and cord blood transplantation are curative treatments for thalassemia, they are not indicated in all patients and are attributed to several complications [17]. Transfusion-dependent thalassemia affects patients' mental health and emotional conditions, causing anxiety and depressive mood disorders. Physical abnormalities, increased risk of death, and activity limitations are responsible for physiological disorders among thalassemia patients [18, 19].

World Health Organization (WHO) defines health as “a state of complete physical, mental and social well-being and not merely the absence of disease or infirmity” [20]. As thalassemia affects patients' physical and psychosocial health, no single physical or physiological index can assess their health. Quality of life (QoL), which comprises physical, psychological, environmental, and social aspects, is an index designed to measure the burden of chronic diseases and evaluate the treatment outcome [21, 22]. There are several questionnaires designed to evaluate the QoL. The World Health Organization Quality of Life Instruments Brief version (WHOQOL-BREF questionnaire) has been proved valid for assessing the QoL across different countries and cultures [23, 24]. Although numerous efforts have been taken to reduce the prevalence and burden of thalassemia in Iran and other parts of the world, thalassemia remains a significant concern [25].

QoL has been shown to be a valid index to assess the overall health in transfusion-dependent thalassemia patients. Although previous studies proved that transfusion-dependent thalassemia patients have relatively lower QoL scores than the normal population, the factors affecting their lower scores are still unknown [26, 27]. This study aims to assess the QoL and its related paraclinical factors in Iranian transfusion-dependent thalassemia patients.

## 2. Methods and Materials

### 2.1. Study Populations

This study is a cross-sectional study performed on 100 patients with transfusion-dependent *ß*-thalassemia at the thalassemia clinic of Imam-Ali Hospital, Karaj, Iran, in 2019. The sample size was estimated by the Lemeshaw formula [28]. Transfusion-dependent thalassemia patients aged more than 18 years old who were not diagnosed with any debilitating disease or complication unrelated to thalassemia are included in the study.

### 2.2. Study Instruments

In this study, we used the WHOQOL-BREF questionnaire in order to assess QoL in patients. The WHOQOL-BREF comprises twenty-six items categorized into four main domains, including physical health (nine facets), psychological state (six facets), social relationship (three facets), and environmental factors (eight facets) (Table 1). The mean score of items within each domain is used to calculate the domain score. Mean scores are then multiplied by four to make domain scores comparable with the scores used in the WHOQOL-100, which is a complete form of WHOQOL-BREF. Domain scores are scaled positively (higher scores are attributed to higher QoL).

### 2.3. Data Collection

Enough time was given to each consented patient to complete the WHOQOL-BREF questionnaire. Also, the patient's demographic and laboratory information, including gender, age, education, marital status, ferritin, fasting blood sugar (FBS), aspartate aminotransferase (AST), Alanine aminotransferase (ALT), and *Z* score, were obtained from their medical record forms. A researcher (MJ) calculated the QoL score based on the WHOQOL-BREF questionnaire guideline. Eventually, another researcher extracted patients' paraclinical indices (MK).

### 2.4. Data Analysis

The collected data were analyzed by SPSS software (v. 22.0, Chicago, IL). Patient characteristics and demographics information were showed by percentage, mean, and standard deviation. QoL scores were presented by the mean and standard deviation. The independent *t*-test was used to find a significant relationship between the clinical and demographics of patients' data. Pearson correlation coefficient was applied to assess the correlation between demographic and clinical variables with QoL domains.

## 3. Results

The demographic patients' characteristics are given in Table 2. 44.2% of participants were male, while 55.8% were female. The patient's mean (±SD) age was 25.78 years (±9.18). It should be noted that 5.7% of participants were illiterate, while 66.7% had primary educations, and 27.6% were university graduates. Most of the participants (*n* = 68, 77.3%) were single.

QoL scores for each domain are presented in Table 3. The patients' physical QoL score was lower compared to all other QoL domains (mean = 12.10, SD = 2.00). While their psychological QoL score was the highest (mean = 13.49, SD = 2.23). Overall, patients acquired higher scores in social domain (mean = 13.46, SD = 3.47) compared to environmental domain (mean = 12.77, SD = 3.01).

Table 4 illustrates the association of domains of QoL (physical, psychological, social, and environmental) and demographic and paraclinical characteristics of included patients using the Pearson correlation coefficient. No significant correlation was seen between QoL domains and age, gender, marital status, and bone marrow densitometry (*Z* score) (*p* value >0.01). While having higher education was correlated with higher phycological and social QoL scores (*p* value <0.01). On the other hand, the ferritin level was negatively correlated with the patient's physical and social scores (*p* value <0.01). Moreover, patients AST and ALT levels were negatively correlated to their physical QoL scores (*p* value <0.01).

Table 5 demonstrates the patients' QoL scores classified by their laboratory findings. Patients with abnormal AST, ALT, and ferritin levels got lower physical QoL scores than patients with normal laboratory findings (*p* value <0.05). Patients with high FBS got significantly lower phycological QoL scores (*p* value <0.05).

## 4. Discussion

Thalassemia, like any other chronic disease, has undeniable effects on different aspects of life. Similar to other studies, we found that the QoL in most thalassemia patients is impaired [1, 29–32]. Dysfunctional daily activities, the need for medical treatment and medications, difficulty in sleeping, pain, and discomfort, fatigability, difficult mobility, and work capacity were all considerable problems in transfusion-dependent thalassemia patients evaluated in the physical domain. The psychological domain considered negative and positive feelings, the ability to concentrate, way of thinking and learning, self-confidence, and acceptance of body appearance, which were all found to be affected by thalassemia. Personal activities, social support, and sexual needs were assessed in the social domain, and all of these fields were affected by the disease. The environment domain evaluated the physical environment, financial resources, freedom, physical safety, and home environment, all of which got low scores in this study.

The patterns of psychological scores WHOQOL-BREF were similar to other previous studies. Although the psychological aspect plays an important role, the symptoms of the illness, the difficulty of the treatment, and repeated absences from school due to hospitalization can significantly impact children's lives. Furthermore, adults with thalassemia deal with additional problems such as finding a partner, making family, and encountering financial problems [30, 33].

Unlike the previous study that reported higher QoL among older patients [1, 34], our study showed no relation between age and QoL. Similar to previous studies, our study revealed that the higher serum ferritin level impacts the QoL. Long-term iron overload and serum ferritin levels higher than 2500 ng/dl are related to cardiovascular issues and mortality [35]. This study focused on patients dependent on transfusion, which can justify some tangible differences compared to previous studies [2].

We demonstrated no relation between age and any of the domains, which is not compatible with other studies [19, 30, 36], which may have been due to the small sample size. Also, our demographic features such as age and marital status are virtually similar among our study population. However, one study showed a significant positive relationship between age and health-related QoL [2].

Similar to the Ansari et al. study, our study also showed a meaningful correlation between the level of education and QoL in patients [1].

Transfusion-dependent thalassemia patients are at a higher risk of endocrine dysfunction such as diabetes mellitus, which can lead to the impaired FBS test and impaired glucose tolerance test (IGT) [37]. Furthermore, studies showed that a high level of FBS is related to lower QoL [38]. This study showed that higher levels of FBS are negatively correlated with psychological QoL in transfusion-dependent thalassemia patients. In addition, recent studies revealed that AST and ALT are higher in thalassemia patients, which indicates the increased risk of liver and heart dysfunction [39]. We also demonstrate that an increased level of AST (more than 42 IU/ml) is related to lower QoL.

There are some questionnaires aiming to evaluate the QoL. One of the valid, reliable, and responsive to change questionnaires is Greek TranQoL. This questionnaire showed acceptable validity and reliability in Greek transfusion-dependent thalassemia patients [40]. It should be noted that Greek TranQoL has not been verified to be used in the Iranian population. On the other hand, WHOQOL-BREF demonstrates excellent validity and reliability in the Iranian population [41]. We believe that using methods such as WHOQOL-BREF questionnaire can help us to evaluate patients' conditions and outcomes. This method is a straightforward and available way that can be utilized to report the impact of the disease in all aspects of patient's lives and help clinicians to prioritize their options in the management of their patients.

One of the limitations of this study was the relatively small sample size. It should be noted that all of the participants were transfusion-dependent. We believe that studies with bigger sample sizes are essential in order to indicate the association of laboratory findings and QoL domains more precisely. Moreover, this study fails to demonstrate the causal relationship between the study parameters. Studies with higher evidence levels should be conducted to evaluate the patients' QoL after improving their laboratory indices.

## 5. Conclusion

The finding of this study demonstrates the impairment of all features of QoL in thalassemia patients. No meaningful relation was noted neither between age and QoL nor between gender and QoL in any domain. Also, marital status and *Z* score did not show any correlation with QoL in any domain. In contrast, education was correlated to the social and psychological domains of QoL. Regarding paraclinical parameters, the ferritin level was related to lower physical QoL and thus may have led to lower QoL in the social aspects of our patient's lives. FBS was associated with lower psychological QoL. AST and ALT both showed a decrease in the physical domain of QoL, and AST also showed a decrease in all domains of QoL. In conclusion, we pointed out some meaningful relations between an inexpensive and straightforward questionnaire and the laboratory checkups that thalassemia patients do on their routine visits. We recommend that this questionnaire be included as part of the routine assessments of thalassemic patients, particularly transfusion-dependent individuals, to help physicians evaluate how patients are feeling and how satisfied they are with their lives' leading to a more effective and efficient treatment.

## 6. Added Value of the Study

This study, to the best of knowledge, for the first time, identified new correlations between QoL and laboratory indices, such as AST, ALT, bone mass densitometry (*z* score), and FBS. In other words, this study proposes novel targets for clinicians and policymakers to assist their patients in order to enhance their QoL.

## Figures and Tables

**Table 1:** WHOQOL-BREF.

Domain	Facets incorporated within domains
(1) Physical health	(1) Activities of daily living (2) Dependence on medicinal substances and medical aids (3) Energy and fatigue (4) Mobility (5) Pain and discomfort (6) Sleep and rest (7) Work capacity

(2) Psychological	(1) Bodily image and appearance (2) Negative feelings (3) Positive feelings (4) Self-esteem (5) Spirituality/religion/personal beliefs (6) Thinking, learning, memory, and concentration

(3) Social relationships	(1) Personal relationships (2) Social support (3) Sexual activity

(4) Environment	(1) Financial resources (2) Freedom, physical safety, and security (3) Health and social care: accessibility and quality (4) Home environment (5) Opportunities for acquiring new information and skills (6) Participation in and opportunities for recreation/leisure activities (6) Physical environment (pollution/noise/traffic/climate) (7) Transport

**Table 2:** Demographic details.

		*N* (%)/mean ± SD
Gender (*n* = 95)	Male	42 (44.2)
	Female	53 (55.8)

Age (*n* = 86)	25.78 ± 9.18	

Education (*n* = 87)	Illiterate	5 (5.7)
	Elementary school	16 (18.4)
	Middle school	32 (36.8)
	Diploma or bachelor of science	10 (11.5)
	Master of science	24 (27.6)

Marital status (*n* = 88)	Not married	68 (77.3)
	Married	20 (22.7)

**Table 3:** Quality of life scores.

Domain	Score (mean ± SD)
Physical	12.10 ± 2.00
Psychological	13.49 ± 2.23
Social	13.46 ± 3.47
Environment	12.77 ± 3.01

**Table 4:** Quality of life scores classified by demographic and clinical characteristics.

	Physical	Psychological	Social	Environment
Gender	0.020	0.120	0.130	0.025
Age	0.015	0.059	0.099	0.124
Education	0.101	0.334^*∗*^	0.328^*∗*^	0.127
Marital status	0.021	0.017	−0.056	0.095
Ferritin	−0.356^*∗*^	−0.067	−0.305^*∗*^	−0.026
FBS	−0.067	−0.353^*∗*^	−0.169	−0.183
AST	−0.356^*∗*^	−0.374^*∗*^	−0.317^*∗*^	−0.370^*∗*^
ALT	−0.319^*∗*^	−0.183	−0.179	0.119
*Z* score	−0.068	−0.068	−0.134	−0.110

^*∗*^Correlation significant at the 0.01 level (2 tailed).

**Table 5:** Quality of life scores classified by laboratory findings.

		Physical	Psychological	Social	Environment
Ferritin	>2500	10.83 ± 1.34	13.30 ± 1.81	10.73 ± 2.44	13.23 ± 2.99
	<2500	12.46 ± 2.02	13.55 ± 2.34	13.36 ± 2.91	13.52 ± 3.60
	*P* value	0.001	0.655	0.001	0.738

FBS	>126	11.72 ± 2.08	12.72 ± 2.62	12.24 ± 2.66	12.68 ± 4.04
	<126	12.26 ± 1.96	13.84 ± 1.95	13.00 ± 3.14	13.81 ± 3.14
	*P* value	0.223	0.024	0.258	0.145

ALT	>56	11.6 ± 1.33	13.14 ± 1.73	12.35 ± 2.58	13.28 ± 3.17
	<56	12.59 ± 2.50	13.90 ± 2.66	13.25 ± 3.40	13.67 ± 3.80
	*P* value	0.026	0.099	0.149	0.587

AST	>40	11.34 ± 1.78	12.51 ± 1.77	11.71 ± 2.70	12.21 ± 3.29
	<40	12.59 ± 2.00	14.14 ± 2.28	13.46 ± 3.02	14.28 ± 3.35
	*P* value	0.003	0.001	0.005	0.004

*Z* score	>−1	12.18 ± 1.97	13.56 ± 2.32	13.05 ± 2.95	13.69 ± 3.54
	<−1	11.80 ± 2.11	13.23 ± 1.89	11.78 ± 3.06	12.66 ± 3.11
	*P* value	0.446	0.552	0.089	0.234

## Data Availability

No data have been submitted to any open-access databases. All the data supporting the study are presented within the manuscript or available upon request.
